# Heteroaryliodonium(III) Salts as Highly Reactive Electrophiles

**DOI:** 10.3389/fchem.2020.599026

**Published:** 2020-11-20

**Authors:** Naoko Takenaga, Ravi Kumar, Toshifumi Dohi

**Affiliations:** ^1^Faculty of Pharmacy, Meijo University, Nagoya, Japan; ^2^J.C. Bose University of Science & Technology, YMCA, Faridabad, India; ^3^College of Pharmaceutical Sciences, Ritsumeikan University, Kusatsu, Japan

**Keywords:** iodine, hypervalent compound, diaryliodonium(III) salt, aromatic substitution, arylation, heteroaromatic compound

## Abstract

In recent years, the chemistry of heteroaryliodonium(III) salts has undergone significant developments. Heteroaryliodonium(III) salts have been found to be useful synthetic tools for the transfer of heteroaryl groups under metal-catalyzed and metal-free conditions for the preparation of functionalized heteroarene-containing compounds. Synthetic transformations mediated by these heteroaryliodonium(III) salts are classified into two categories: (1) reactions utilizing the high reactivity of the hypervalent iodine(III) species, and (2) reactions based on unique and new reactivities not observed in other types of conventional diaryliodonium salts. The latter feature is of particular interest and so has been intensively investigated in recent decades. This mini-review therefore aims to summarize the recent synthetic applications of heteroaryliodonium(III) salts as highly reactive electrophiles.

## Introduction

Over the past few decades, the hypervalent iodine(III) reagent has been widely recognized as a versatile oxidant for performing environmentally friendly oxidation reactions. It has gained great importance as a promising alternative to toxic heavy metal oxidants, such as Pb(IV), Tl(III), and Hg(II) because of its low toxicity, high stability, and easy handling (Stang and Zhdankin, 1996; Zhdankin and Stang, 2002, 2008; Zheng et al., 2014; Yoshimura and Zhdankin, 2016). Diaryliodonium salts (Ar^1^Ar^2^I^+^X^−^), which have two aryl groups and one counter-anion on the trivalent iodine atom, represent one of the most useful classes of hypervalent iodine(III) compounds (Merritt and Olofsson, 2009; Yusubov et al., 2011; Aradi et al., 2016; Fañanás-Mastral, 2017; Stuart, 2017). Due to the highly electron-deficient nature of the iodine(III) center in diaryliodonium salts and the excellent leaving-group ability of the iodobenzene group, they serve as highly reactive arylating agents toward a wide range of nucleophiles, even under metal-free conditions. Although the chemistry of conventional diaryliodonium salts is well developed and widely applied, synthetic transformations using heteroaryliodonium salts have received little attention to date.

In early studies, symmetric diaryliodonium salts (Ar^1^Ar^2^I^+^X^−^; Ar^1^ = Ar^2^) were preferred over unsymmetric salts (Ar^1^Ar^2^I^+^X^−^; Ar^1^ ≠ Ar^2^) to avoid selectivity issues during the aryl-transfer processes. Meanwhile, the introduction of a “dummy aryl” into asymmetric salts to act as a nontransferable group has been favored in recent studies to modulate and control the reactivities of the diaryliodonioum salts. On the other hand, the reactions of heteroaryliodonium salts have some limitations in the past; for example, the heteroaryl group present in phenyl(heteroaryl)iodonium salts tends to serve as a “dummy aryl,” and it was not possible to transfer the heteroaryl group during the reactions (Martín-Santamaría et al., 2000). However, during the last decade or so, quite interesting and highly valuable transformations utilizing aryl(heteroaryl)iodonium salts have been reported. Thus, this mini-review highlights the representative examples in both metal-catalyzed and metal-free reactions based on the currently available literature.

## Heteroaryliodonium(III) Salts: Preparation

**Figure 1:**
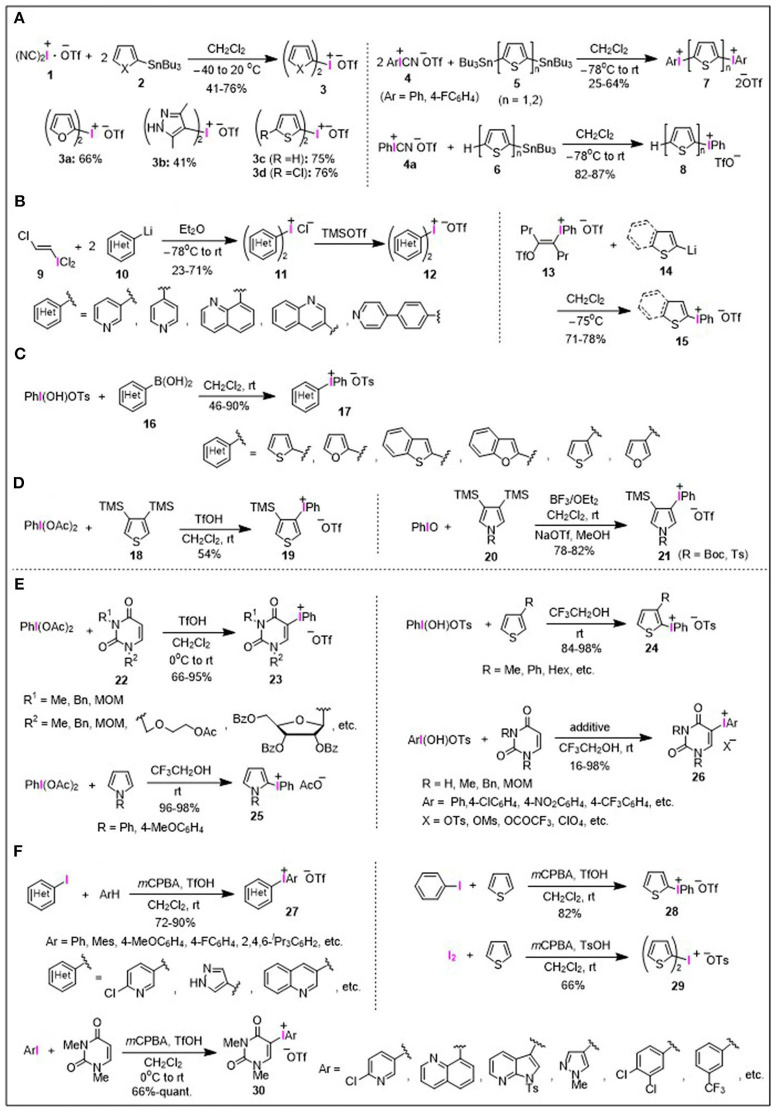
Synthesis of heteroaryliodonium salts. **(A)** Stannyl heteroarenes. **(B)** Lithio heteroarenes. **(C)** Boryl heteroarenes. **(D)** Silyl heteroarenes. **(E)** Direct synthesis. **(F)** One-pot synthesis from iodoarenes.

The conventional synthetic routes to heteroaryliodonium salts rely on stepwise processes using a series of organometallic nucleophiles instead of heteroarenes themselves, as outlined in Figures 1A–D. One general method for the preparation of heteroaryliodonium salts is based on the iodonium(III)-atom transfer of iodine(III) cyanides to heteroarylstannanes (Figure 1A). More specifically, (dicyano)iodonium(III) triflate [(NC)_2_I^+−^OTf] **1**, generated *in situ* from iodosyl triflate and trimethylsilyl cyanide (TMSCN), reacts with tributyltin precursors **2** derived from furans, thiophenes, and pyrazoles, to afford the corresponding symmetrical bis(heteroaryl)iodonium triflates **3a**–**3d** (Figure 1A; Stang et al., 1992a,b). Aryl(cyano)iodonium(III) triflates (ArI^+^CN^−^OTf) **4** could also be used as an iodine transfer agent, reacting with stannylated derivatives **5** and **6** to afford a variety of mixed thienyl(aryl)iodonium salts **7** and **8**, respectively (Figure 1A; Gallop et al., 1993; Zhdankin et al., 1993; Bykowski et al., 2002).

The iodonium(III)-transfer procedure using *trans*-(chlorovinyl)iodonium dichloride **9** (Beringer and Nathan, 1969) was reported to access the bis(heteroaryl)iodonium chlorides **11** by the reaction of lithiated nitrogen-containing heteroarenes **10** (Figure 1B; Stang and Chen, 1995; Stang et al., 1995). Thus, the prepared bis(heteroaryl)iodonium chlorides **11** could be converted to the corresponding triflates **12** by treatment with trimethylsilyl triflate (TMSOTf). For the preparation of unsymmetrical heteroaryl(phenyl)iodonium triflate **15**, the ligand-transfer reaction between vinyliodonium salt **13** with heteroaryllithium **14** was developed by Kitamura et al. (Figure 1B; Kitamura et al., 1996).

Another approach to unsymmetrical heteroaryl(phenyl)iodonium salts is the combination of readily available heteroarylboronic acids **16** with hydroxy(tosyloxy)iodobenzene (PhI(OH)OTs; Koser's reagent), which is useful for the mild and regioselective preparation of tosylate salts **17** (Figure 1C; Carroll et al., 2000).

Silylated heteroarenes can also be utilized for the preparation of heteroaryliodonium salts; phenyliodonium triflate **19** was prepared from bis(trimethylsilyl)thiophene **18** by the reaction with phenyliodine diacetate [PhI(OAc)_2_; PIDA] and triflic acid (Ye et al., 1996), while pyrrolyliodonium triflate **21** was obtained by the reaction of bis(trimethylsilyl)pyrroles **20** with iodosobenzene (PhIO) in the presence of BF_3_•Et_2_O (Figure 1D; Liu et al., 1999).

Straightforward approaches for the direct/one-pot preparation of heteroaryliodonium salts from heteroarenes have been elaborated as shown in Figures 1E,F, which allow expansion of the substrate scope to provide diverse types of heteroaryliodonium salts. In contrast to the aforementioned iodonium(III) salts containing fundamental heteroarene units, such as thiophene, pyridine, and furan, iodonium(III) salts bearing nucleobase and nucleoside moieties did not appear in the literature until 1998. Kim et al. reported the preparation of phenyliodonium(III)-substituted uracil nucleosides **23** by the reaction of uracil nucleosides **22** with PIDA in the presence of triflic acid, and its subsequent application in palladium-catalyzed alkenylation reaction (Figure 1E; Roh et al., 1998, 1999). Utilizing the unique character of the fluoroalcohol medium as a solvent, Kita et al. developed a facile synthesis of heteroaryliodonium salts **24** and **25** through the condensation of thiophenes and pyrroles with an iodine(III) reagent, whereby trifluoroethanol (TFE) enhanced the efficiency of this type of condensation, significantly extending the product scope (Dohi et al., 2007, 2010; Ito et al., 2010). Furthermore, this procedure could also be applied to the preparation of uracil-aryliodonium salts **26** possessing different types of aryl moieties and various counterions (Takenaga et al., 2018, 2019a).

Moreover, Olofsson et al. developed an efficient one-pot synthesis to heteroaryliodonium triflates **27** and **28** from arenes and aryl iodides using *m*-chloroperbenzoic acid (*m*CPBA) as a stoichiometric oxidant in the presence of triflic acid. In a seminal report, heteroaryliodonium tosylates could be prepared using *p*-toluenesulfonic acid instead of triflic acid as an additive (Figure 1F; Bielawski and Olofsson, 2007; Bielawski et al., 2007, 2014). Symmetrical di(heteroaryl)iodonium salt **29** was synthesized *via* a modified one-pot procedure employing elemental iodine, arenes, *m*CPBA, and toluenesulfonic acid under similar conditions (Figure 1F; Zhu et al., 2008). The one-pot procedure was also applied to the preparation of uracil-aryliodonium salts **30** bearing various aryl moieties (Toh et al., 2013; Modha and Greaney, 2015). As a result, recent progress in the efficient synthesis of diaryliodonium(III) salts has enabled the facile preparation of diverse types of heteroaryliodonium salts.

## Synthetic Applications

**Figure 2:**
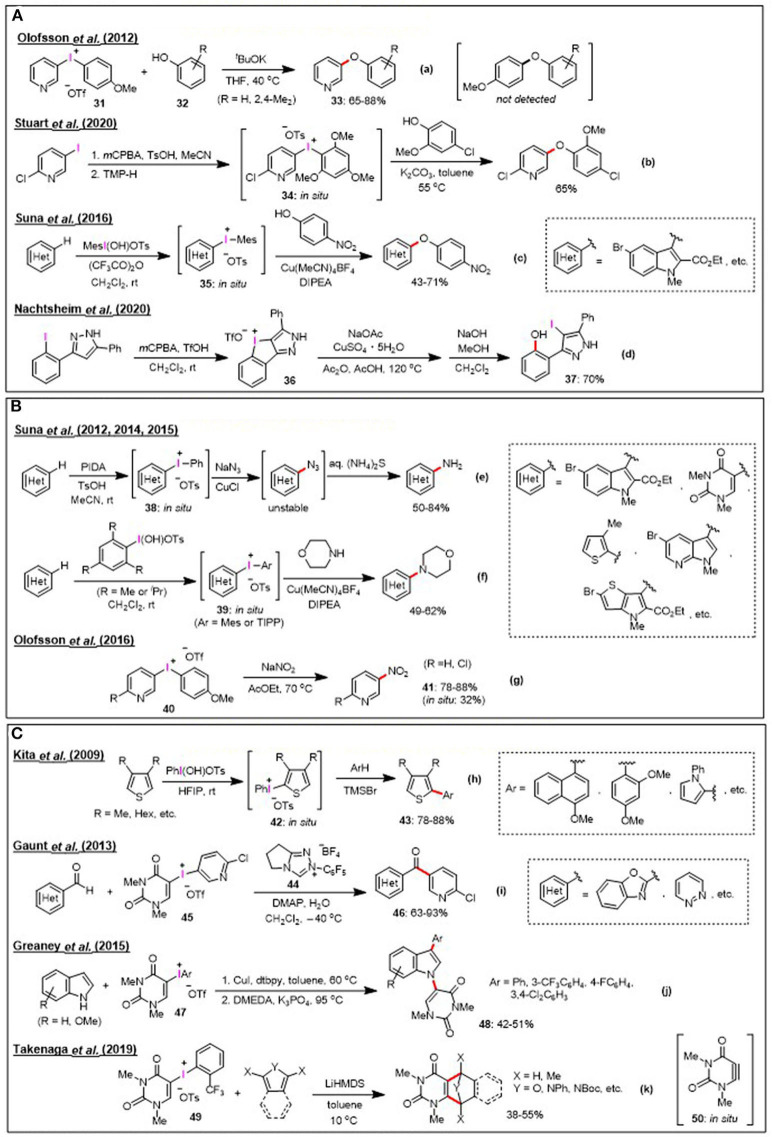
New synthetic application of heteroaryliodonium salts. **(A)** C-O bond formation [metal-free: (a,b), metal-catalyzed: (c,d)]. **(B)** C-N bond formation [metal-catalyzed: (e,f), metal-free: (g)]. **(C)** C-C bond formation [metal-free: (h,i), metal-catalyzed: (j), aryne analog precursor: (k)].

Various organic synthetic transformations utilizing diaryliodonium salts have emerged over the past few decades, and this research field includes diverse metal-catalyzed and metal-free arylations for a wide range of nucleophiles, in addition to benzyne generation and the dearomatization of phenols. In addition to these reactions, new synthetic applications of heteroaryliodonium salts have been investigated in recent years. Herein, we outline the strategies based on the use of heteroaryliodonium(III) salts as electrophilic aryl transfer reagents for the C-O, C-N, and C-C bond formations. The synthetic transformations of heteroaryliodonium salts are classified into the following two categories: (1) reactions utilizing the high reactivities of iodonium(III) salts, and (2) reactions based on the unique and new reactivities of heteroaryliodonium salts. The former category of transformation has been applied to C-O and C-N bond formation reactions (Figures 2A,B), while the latter has been employed in C-C bond formation processes (Figure 2C).

### Dummy Groups for Selective Aryl Transfer From Diaryliodonium Salts

Over the past few decades, symmetrical heteroaryliodonium salts (Ar^1^Ar^2^I^+^X^−^; Ar^1^ = Ar^2^) have found numerous applications as electrophilic arylating agents for nucleophiles. In contrast, unsymmetrical salts (Ar^1^Ar^2^I^+^X^−^; Ar^1^ ≠ Ar^2^) have been less frequently employed since the presence of two different aromatic moieties in the salts could potentially give mixtures of two arylated products (i.e., Ar^1^-Nu and Ar^2^-Nu). Recently however, by the introduction of a nontransferable dummy group, unsymmetrical iodonium salts participate in a wide range of transformations. The representative examples of dummy groups are *p*-anisyl (Ma et al., 2015; Xiong et al., 2015), mesityl (Matsuzaki et al., 2015; Sundalam and Stuart, 2015), and 2,4,6-trimethoxyphenyl (TMP) (Malmgren et al., 2013; Seidl et al., 2016) groups, and these dummy groups were utilized in reactions of the conventional diaryliodonium salts.

### Heteroaryliodonium Salts as Highly Reactive Pseudo-Halides

Selected examples of C-O bond formation reactions by the electrophilic arylation of phenols using heteroaryliodonium salts are shown in Figure 2A, Equation a–c. In such cases, phenols were efficiently arylated to afford industrially important aryl(heteroaryl) ether compounds. In 2012, Olofsson et al. studied the metal-free arylation of phenols under basic conditions. They confirmed the chemoselective reactivity pattern of unsymmetrical diaryliodonium salts (Jalalian et al., 2012; Bielawski et al., 2014) (Figure 2A, Equation a). It was revealed that the electronic effect of the aryl rings was dominant during the reaction of *p*-anisyl (pyridyl) triflate **31** with phenols **32**; the phenol nucleophiles selectively coupled to the electron-deficient pyridyl group to yield pyridyl ether **33** in 65–88% yield. This chemoselectivity trend has been further applied by other researchers to transfer the electron-poor aryl group of unsymmetrical iodonium salts, and it was noted that the TMP group serves as an improved dummy ligand for aryl transfer processes from heteroaryl(TMP)iodonium salts (Seidl et al., 2016; Dohi et al., 2017). In 2020, Stuart et al. reported a convenient method for phenol arylation using the *in-situ*-generated aryl(TMP)iodonium salts **34** (Gallagher et al., 2020) (Figure 2A, Equation b). Thus, in metal-free reactions, nucleophiles would preferentially react with the more electron-deficient aromatic ring out of the two aryl moieties in unsymmetrical diaryliodonium salts. The results are however different in case of metal-catalyzed reactions. Suna et al. demonstrated that the presence of a Cu(I) catalyst directed the selectivity of the arylation to the more electron-rich heteroarene moiety during the reaction between heteroaryliodonium salts **35** and phenols (Sokolovs and Suna, 2016) (Figure 2A, Equation c). This Cu(I)-catalyzed synthesis of diaryl ethers using diaryliodonium salts is a complementary strategy to the previously mentioned metal-free arylation of phenols. The arylation of oxygen nucleophiles using heteroaryliodonium salts was not limited to the preparation of aryl(heteroaryl) ethers; as in the cases of other oxygen nucleophiles, it was demonstrated that benzoic acid was also arylated to produce quinolin-3-yl benzoate *via* the *in-situ*-generated iodonium salt bearing a 3-quinolyl group (Dohi et al., 2017). Further, the chemistry of cyclic iodonium salts has recently experienced growing development (Chatterjee and Goswami, 2017). As for just an emerging interesting compound, Nachtsheim et al. investigated that the direct oxidative cyclization of 3-(2-iodophenyl)-1*H*-pyrazole to novel iodolopyrazolium salt **36** *via in-situ* formed *N*-heterocyclic stabilized hydroxy- λ^3^-iodane (Boelke et al., 2020) (Figure 2A, Equation d). They also demonstrated that the copper-catalyzed ring opening of **36** with acetate followed by hydrolysis produced functionalized heteroaromatic biaryl **37** in 70% yield.

Various heteroaryliodonium salt-mediated C-N bond formation processes are summarized in Figure 2B; these reactions include the azidation, amination, and nitration of heteroarenes. In 2012, Suna et al. reported the Cu(I)-catalyzed transformation of C–H to C–N in electron-rich heteroaromatics (i.e., pyrroles, pyrrolopyridines, thienopyrroles, pyrrolopyrimidines, and uracil), which involves the reaction of *in-situ* prepared heteroaryl(phenyl)iodonium tosylates **38** with sodium azide, whereby complete regiocontrol is accomplished (Lubriks et al., 2012) (Figure 2B, Equation e). Based on this study, they further demonstrated the C–H amination of electron-rich heteroarenes, comprising the *in-situ* formation of heteroaryliodonium salts **39** bearing sterically hindered mesityl (Mes) or triisopropylphenyl (TIPP) group (Sokolovs et al., 2014; Berzina et al., 2015) (Figure 2B, Equation f). The electronic effects observed for the aryl transfer are consistent with those mentioned earlier for *O*-nucleophiles. Thus, the electron-rich heteroarene moieties of these salts were transferred in Cu(I)-catalyzed arylations, while *N*-nucleophiles were preferentially coupled with the electron-deficient heteroaromatic ring under metal-free conditions. Sodium nitrite was recently used as a nucleophile for diaryliodonium salts as a convenient approach to access synthetically useful nitroarenes. In 2016, Olofsson et al. reported the metal-free nitration of *p*-anisyl(pyridyl) salt **40** to afford nitropyridine **41** (Reitti et al., 2016) (Figure 2B, Equation g). Other metal-free reactions with *N*-nucleophiles include application of the TMP group as an extremely electron-rich aryl dummy ligand to transfer electron-deficient pyridyl groups to alicyclic amines or the azide ion (Sandtorv and Stuart, 2016; Seidl and Stuart, 2017).

### Unique Reactions Based on the Use of Heteroaryliodonium Salts

Figure 2C lists the versatile C-C bond forming reaction based on the use of heteroaryliodonium salts as aryl sources and reaction intermediates. For example, in 2009, Kita et al. demonstrated the metal-free oxidative cross-coupling of heteroarenes with electron-rich arenes by direct C–H transformations. The reaction proceeded through the *in-situ* formation of thienyliodonium salt **42** from an electron-rich thiopene and Koser's reagent in hexafluoroisopropanol (HFIP). It was proposed that the intermediate thienyliodonium species was activated by the addition of trimethylsilyl bromide, followed by its coupling with electron-rich arenes to provide a wide range of useful mixed biaryl products **43** (Kita et al., 2009, 2011; Morimoto et al., 2010, 2011; Dohi et al., 2013) (Figure 2C, Equation h). In 2013, Gaunt et al. developed a new acyl coupling reaction that merges the aryl transfer ability of diaryliodonium salts with carbonyl umpolung using an *N*-heterocyclic carbene (NHC) catalyst **44**. They also established the transfer of a pyridyl group from several pyridyliodonium salts to provide di(hetero)aryl ketones **46**, and exclusive pyridyl transfer was observed when a uracil-derived salt **45** was used. This is an interesting example of utilization of the uracil moiety as a nontransferable dummy group for the organocatalytic reactions of diaryliodoniuim(III) salts, with more frequently used dummy groups for arylations being the Mes, TMP, and TIPP groups (Toh et al., 2013) (Figure 2C, Equation i). In general, arylation using diaryiodonium salts produces one equivalent of iodoarene as a waste byproduct, which is problematic in the context of atom economy. Thus, Greaney et al. reported the very atom-economical transformation of heteroaryliodonium salts (Modha and Greaney, 2015) (Figure 2C, Equation j). They showed that uracil-iodonium salts **47** could undergo Cu-catalyzed tandem C-H/N-H arylation reactions to produce double-arylated indoles **48** by incorporation of both the uracil and aryl groups. As another copper-catalyzed tandem reaction, Fañanás-Mastral et al. described an interesting example, carboarylation/cyclization of alkynyl phosphonate with a mesityl thienyl iodonium salt (Pérez-Saavedra et al., 2017). The recent report by Takenaga et al. highlighted the synthesis of bicyclic uracil systems and the vicinal functionalization of uracils with the basic activation of salts **49** being stabilized by the *o*-trifluorophenyl group. The reactive intermediate of this reaction is believed to involve the generation of a highly strained heterocyclic alkyne species, uracilyne **50**, a non-reported heteroaryne analog (Takenaga et al., 2019a,b) (Figure 2C, Equation k).

## Conclusion and Outlook (Future Perspectives)

In the twentieth century, the synthetic scope of heteroaryliodonium(III) salts was limited, and thus only relatively simple heteroaryliodonium salts, such as symmetrical bis(heteroaryl)iodonium salts and unsymmetrical phenyl(heteroaryl)iodonium salts, were previously reported. With recent advances in efficient synthetic procedures, heteroaryliodonium salts bearing sterically hindered mesityl, trimethoxyphenyl, and triisopropylphenyl groups were efficiently synthesized. These designer heteroaryliodonium salts have been found to control the chemoselectivity of aryl transfer in a wide range of reactions. The metal-free and metal-catalyzed reactions show significant selectivity toward aryl/heteroaryl group transfer and unearth an important synthetic methodology for selective synthetic purposes. The synthetic utilities of heteroaryliodonium salts highlighted in this mini-review, such as NHC-catalyzed C-H bond arylation, Cu-catalyzed tandem arylation of indoles, and vicinal functionalization, undoubtedly pave a useful pathway in this direction, and developments of new synthetic transformations in this area are highly demanded in future.

## Author Contributions

NT and TD conceived and wrote the manuscript. All authors provided comments, discussed the manuscript, and approved it for publication.

## Conflict of Interest

The authors declare that the research was conducted in the absence of any commercial or financial relationships that could be construed as a potential conflict of interest.
